# The Constructions and Pyrolysis of 3D Kerogen Macromolecular Models: Experiments and Simulations

**DOI:** 10.1002/gch2.201900006

**Published:** 2019-04-07

**Authors:** XiaoHe Wang, XianFu Huang, Kui Lin, Ya‐Pu Zhao

**Affiliations:** ^1^ State Key Laboratory of Nonlinear Mechanics Institute of Mechanics Chinese Academy of Sciences Beijing 100190 China; ^2^ School of Engineering Science University of Chinese Academy of Sciences Beijing 100049 China

**Keywords:** chemical kinetics, hybrid MD/fbMC approach, kerogen, macromolecular models, pyrolysis

## Abstract

Kerogens are extracted from deep shales to study pyrolysis of deep shale samples. The 2D molecular models of kerogens are obtained by a series of physical and chemical experiments by which the macromolecular models of kerogens are constructed. Then, the reasonable 3D macromolecular models are established by molecular mechanics and global energy minimization. The effects of temperature and heating rate on the chemical kinetics of kerogen pyrolysis are studied using reactive force field (ReaxFF). The hybrid molecular dynamics/force‐biased Monte Carlo (MD/fbMC) approach is used to simulate the pyrolytic process at the experimental temperature, which is lower than the conventional one. The gaseous products and residues obtained by the simulations agree with the experimental results, which means a reliable simulation method for pyrolysis at experimental temperature is provided. This study constructs the rational macromolecular models of kerogen by experiments, and proposes the mechanisms of typical reactions of kerogen pyrolysis, which may help in understanding the formation of shale oil and gas.

## 1 Introduction

**Figure 1:**
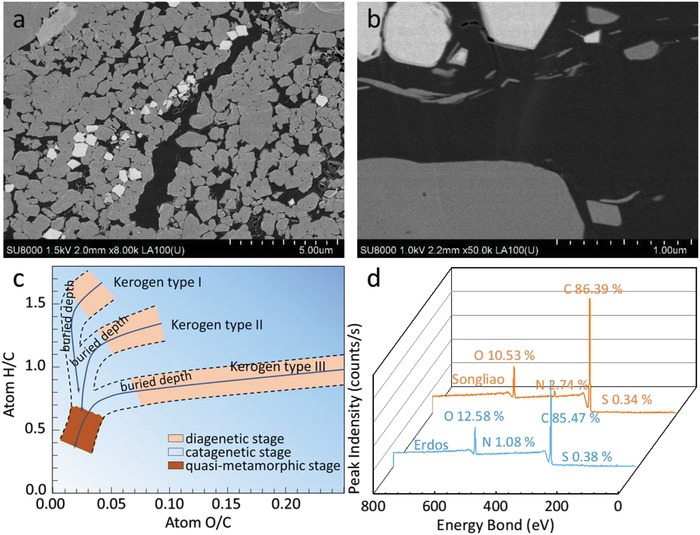
a) SEM image of shale. b) SEM image of kerogen and organic porosity. c) Van Krevelen diagram of the evolution stages and classification of kerogen types. d) The XPS spectra of Songliao and Erdos kerogens.

Nowadays, the development of shale oil and gas has brought tremendous changes to the pattern of world energy. The study of the oil/gas‐forming mechanism of organic matter cracking into shale oil/gas is of great significance to the shale gas exploration, the production forecast and efficient production. Kerogen is the main organic matter and gas‐generating/oil‐forming parent material in sedimentary rocks. It has undergone thermal evolution and microbial transformation to form shale oil/gas over geological time. The shale oil/gas mainly exists in the form of associative adsorption in the rock and organic pores surface, and partly in the form of dissociative adsorption and free state.^1^ A complex structure is formed in shale reservoirs with a large number of micro‐nanopores in the range of 2–50 nm by small‐angle scattering, focused ion beam/scanning electron microscopy (FIB/SEM) and adsorption (**Figure** **1**a,b).^2^ Shale oil/gas are confined to the micro‐nanopores of rock with the changing reservoir pressure, and adsorption phase transition occurs.^3^, ^4^ The studies of the kerogen molecular structure and the hydrocarbon generation can help to elucidate the organic matter‐generating oil/gas potential, and clarify the mechanism of oil/gas outward transportation from organic matter.^5^ However, the chemical morphology, mechanical properties and oil/gas generation mechanism of kerogen are still unknown, as kerogen has an amorphous structure and it is difficult to separate from shale and insoluble in common solvents.^6^

The kerogen classification defined by van Krevelen diagram is based on hydrogen/carbon (H/C) and oxygen/carbon (O/C) ratio as shown in Figure 1c. Type I kerogen (H/C > 1.25, O/C < 0.15), mainly enriched in lacustrine sedimentary rock and containing alkanes, aromatic compounds, and a small amount of oxygen functional compounds, is the most important type of oil and gas production. Type II kerogen (H/C < 1.25, O/C = 0.03–0.18) mainly comes from the ocean plankton and exists in the marine sediments, which has good oil and gas production capacity but lower than that of type I kerogen. Type III kerogen (H/C < 1, O/C = 0.03–0.3), mainly from higher plants with low hydrogen content, is with a large number of cyclic and aromatic compounds in kerogen (oil production capacity of type III kerogen is relatively low, but it is the main type of gas production when the shale is with deep burial).^7^

The understanding of kerogen is mainly based on the2D model of large complex carbon molecules or 3D molecular structure described by molecular dynamics (MD) simulation. Physical and chemical degradation experiments are the main methods to study the kerogen structures. The elemental analysis (EA), X‐ray photoelectron spectroscopy (XPS), Fourier transform infrared spectroscopy (FT‐IR), and ^13^C solid‐state nuclear magnetic resonance (^13^C NMR) are applied to measure the compositions of elements, the elemental chemical state, types and relative abundances of functional groups in kerogen.^8^, ^9^ And the micromolecular information contained in a kerogen molecule is analyzed by pyrolysis gas‐chromatogram and mass‐spectrogram (PY‐GC/MS).^10^, ^11^, ^12^ The nuclear magnetic spectra of mudstone and xylogen (the common nonhydrocarbon compounds of kerogen) have been obtained by ^13^C NMR.^13^, ^14^ Coal structures with the high, medium, and low coal ranks have been built by XPS, ^13^C NMR, and EA which have been used in the study of kerogen molecular constructions.^15^, ^16^ The Kimmeridge kerogen molecular formula has been constructed from the mild oxidative degradation by ruthenium tetroxide. The 2D kerogen macromolecule models of Estonian shale and Green River oil shale have been established by experimental methods.^8^, ^17^

The models of type I kerogen of Green River, type II kerogen of Paris basin, and type III kerogen of Cameroon have been built, which belong to different evolution stages (diagenetic stage, catagenetic stage, and quasi‐metamorphic stage).^18^ The 3D molecular models have been established by MD simulations.^18^, ^19^ Then the method has been applied to the establishment of 3D kerogen molecules, and the 3D model of Siskin Green River kerogen has been build based on ab initio and MD simulation.^17^ A set of “real” molecular models of kerogen have been developed using an experimental‐simulation approach.^20^ Shale gas is likely to adsorb on the wall of the carbon skeleton of kerogen. Therefore, the molecular structure of kerogen is closely related to the adsorption and transport of shale gas.^20^, ^21^ In addition, the maturity of kerogen is another factor that relates to its gas adsorption capacity. The higher the maturity of kerogen is, the bigger its gas adsorption capacity is.^22^ The construction of realistic molecular model of kerogen is the foundation to study the oil/gas generation, adsorption/desorption, and transport of gas molecules in the its nanopores, and therefore need further researches.

The connectivity between atoms in the molecule of the reactive force field (ReaxFF) is determined by the interatomic bond order at that time, which means the chemical bond can be broken and formed freely, so that the chemical reaction process can be obtained.^23^, ^24^ The ReaxFF method has been successfully applied to reaction kinetics simulation researches, including applications of the organic micromolecular system, the polymer system, and the high energy material system.^23^, ^24^, ^25^, ^26^, ^27^, ^28^, ^29^, ^30^ The result of ReaxFF method has been verified to be consistent with that of the quantum mechanics (QM) method and the experiment.^27^ In order to enable chemical reactions occurring in computational time which is much smaller than the experimental time scale, ReaxFF simulations take temperatures higher than that of experiments. Previous studies have confirmed the feasibility and accuracy of ReaxFF simulation of coal pyrolysis.^31^, ^32^, ^33^, ^34^ Then the ReaxFF method has been extended and applied to the pyrolytic simulation of kerogen.^35^ However, the reaction process and intermediate products of hydrocarbon generation are rarely reported, and the simulation temperature is much higher than experimental temperature.

In this study, kerogen macromolecules (large enough) containing the functional groups that are very few in kerogen, have been established by means of physical and chemical experiments. 3D molecular models are built by MD simulations by which the pyrolytic process and the composition of kerogen pyrolysis products under different temperature control are studied using ReaxFF. The new approach of hybrid molecular dynamics/force‐biased Monte Carlo (MD/fbMC) is used for kerogen pyrolytic simulations at experimental temperatures. The results of simulations and experiments are in good agreement. Our findings may expand our knowledge of kerogen pyrolysis and assist to further study of relationships of kerogen pyrolysis to the kerogen pore structure and oil/gas transportation.

## 2 Results and Discussion

### 2.1 Constructions of Kerogen Macromolecular Models

#### 2.1.1 Classification of Kerogen Samples

As shown in **Table** **1** and Figure 1d, the results of XPS and EA indicated that the H/C atom ratios of two types of kerogen are 1.40 and 0.85, the O/C ratios are about 0.02–0.12 and 0.1–0.15, respectively. The two kerogen types of Erdos and Northeast China can be assigned to type I and type III kerogen, respectively, by the van Krevelen diagram.

**Table 1:** The results of elemental analysis

Type	N %	C %	H %	H/C [atom ratio]	N/C [atom ratio]
Songliao	2.62	74.75	8.70	1.40	0.03
Erdos	1.24	36.38	2.90	0.85	0.03

#### 2.1.2 Existence of Kerogen Elements by XPS

**Figure 2:**
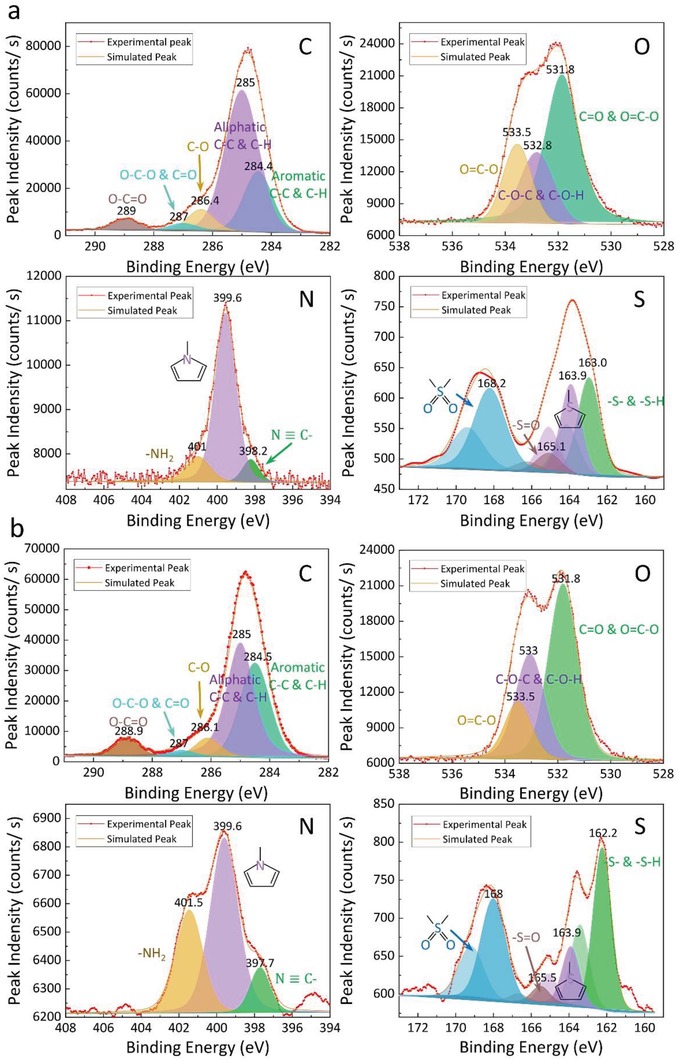
The chemical states and their content analysis of major elements in a) Songliao kerogen and b) Erdos kerogen by XPS. The fitting peaks of corresponding chemical states were filled by different colors. The red curves are the experimental results. The orange curves are the simulated peaks.

The XPS spectra were divided into several fitting peaks to calculate the relative contents of chemical states, because there are one more chemical states of these elements.^34^, ^35^, ^36^ The fitting peaks filled by different colors correspond to different chemical states of elements which are shown in **Figure** **2** and **Table** **2**. And the simulated peaks (orange curve) are obtained by summing the fitting peaks up. As shown in Figure 2, the agreement between simulated peak points and the experimental peak points is excellent, which indicates that the peak splitting of XPS spectra is suitable. Subsequently, the peak positions and corresponding fractions of areas of each fitting peak were calculated to obtain the relative abundances of different chemical states of each element. The main chemical states and their relative contents of C, O, nitrogen (N), and sulfur (S) are listed in Table 2.^16^, ^36^, ^37^ The Erdos kerogen contains more aromatic groups than Songliao kerogen, which will be compared to the result of ^13^C NMR later. The atomic ratios of C, O, N, and S, and the relative percentages of chemical states are obtained to construct molecular models can be acquired.

**Table 2:** The chemical states percentage of Songliao and Erdos kerogens elements

Element	Chemical states	Binding energy [Songliao]	Percentage [Songliao]	Binding energy [Erdos]	Percentage [Erdos]
C 1s	Hydrocarbon (Aromatic)	284.446	23.23	284.500	37.38
	Hydrocarbon (Aliphatic)	285.000	63.70	285.000	48.64
	Ether, Alcohol	286.391	7.14	286.100	6.31
	Carbonyl, O–C–O	287.000	2.22	287.000	1.80
	Ester, carboxyl	288.962	3.71	288.862	5.87
O 1s	Ester, Carbonyl	531.854	57.24	531.800	52.72
	Ether, hydroxy	532.800	22.13	533.051	31.47
	Ester, carboxyl	533.541	20.63	533.500	15.81
N 1s	Nitrile	398.224	8.86	397.700	56.93
	Pyrrole	399.558	77.52	399.600	32.59
	Amino	401.005	13.62	401.450	10.48
S 2p	Mercaptan, Thioether	162.960	31.46	162.239	44.28
	Thiophene	163.922	25.53	163.898	15.26
	Sulfoxide	165.086	5.94	165.509	4.35
	Sulfone	168.219	37.07	168.000	36.11

#### 2.1.3 Functional Groups of Kerogens by FT‐IR

**Figure 3:**
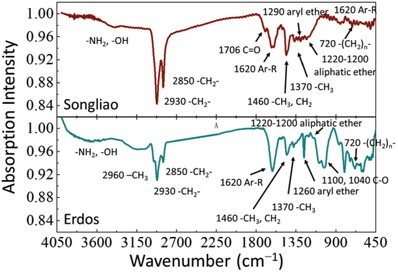
The functional groups of kerogens obtained by FT‐IR.

The location and relative intensity of the absorption bands of FT‐IR spectra reflect the functional group composition, relative abundance, and bond properties of the kerogen. As shown in **Figure** **3**, the kerogen is composed of aliphatic structures, aromatic structures, and heteroatomic functional groups whose main component is oxygen‐containing functional groups. In conjunction with Table S1 in the Supporting Information, the kerogen functional groups reflected by the vibration frequencies are shown in Figure 3. The stretching vibration absorption bands of methyl and methylene are near 2960, 2920, and 2850 cm^−1^ in the FT‐IR spectra. The band near 1600 cm^−1^ represents the characteristic peak of aromatic ring, and the wavenumber of ether is in 1300–1000 cm^−1^. The –(CH_4_)_n_‐ (n > 4) group is represented by characteristic peak near 720 cm^−1^. The fingerprint region (880–680 cm^−1^) of phenyl ring substitution, containing many characteristic peaks, indicates the samples have multiple substitutions. As shown in Figure 3, the spectra of two kerogen types have enormous difference. Songliao kerogen is with strong absorption peaks located in the region of 2850–3050 cm^−1^, and the absorption peak of aromatic ring is weaker than that of saturated carbon bonds. While the infrared spectrum of Erdos kerogen has strong absorption peaks of aromatic, Songliao kerogen contains more aliphatic functional groups and less oxygen‐containing functional groups than Erdos kerogen.

#### 2.1.4 Kerogen Molecular Carbon Skeleton by ^13^C NMR

**Figure 4:**
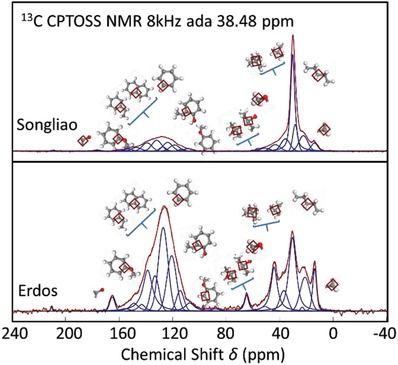
The ^13^C NMR spectra of kerogen and their overlapping peak resolving. The magenta lines, the purple lines, and the maroon lines represent the fitting peaks of different carbon‐containing functional groups, the simulated peaks, and the experimental results reporting the ^13^C NMR spectra, respectively.

The ^13^C NMR spectra of kerogens are with mainly three bands of kerogens: the aliphatic regions at 0–90 ppm, the aromatic regions at 100–165 ppm, and carbonyl carbon regions at 165–220 ppm. As the proportions of areas in **Figure** **4** represent the abundances of different carbon components, Songliao kerogen contains many aliphatic carbons, while aromatic carbon is the main component in Erdos kerogen. Both samples have the smallest amount of hydroxy carbon, while the hydroxy carbon of Erdos kerogen is more than that of Songliao (it is consistent with the results of XPS and EA). Overlapping peak resolving of the ^13^C NMR spectrum is needed to obtain the fitting peaks, because the samples contain several functional groups. The fitting peaks at the corresponding chemical shift are shown in Figure 4, and the simulated peaks can be obtained by summing the fitting peaks up. In order to get reasonable carbon skeleton, the simulated peaks were compared to the entire experimental ^13^C NMR spectra and then parameters of the fitting peaks were adjusted, until the simulated peaks coincided with the experimental peaks. The fitting spectra and the experimental spectra are fitted very well as shown in Figure 4, which indicates the peak splitting of ^13^C NMR spectrums are suitable. The relative areas of fitting peaks, representing the abundance of the carbon‐containin functional groups, are listed in **Table** **3**.^8^, ^38^

**Table 3:** The carbon skeletons of Songliao and Erdos kerogens

Kerogen sample		Aliphaticity					
		Methyl $\left[f_{\mathrm{al}}^{1}\right]$	Methylene $\left[f_{\mathrm{al}}^{2}\right]$	Methine/Quaternary $\left[f_{\mathrm{al}}^{3}\right]$	Oxy‐Methylene $\left[f_{\mathrm{al}}^{O_{1}}\right]$	Oxy‐Methine/Oxy‐quaternary $\left[f_{\mathrm{al}}^{O_{2}}\right]$	Carboxyl/Carbonyl
Songliao	Chemical Shift (PPM)	14.41	22.68/28.65/30.76	36.21/43.76	51.51/57.33	61.12	177.41/220.26
	Relative area	0.038	0.52	0.11	0.024	0.001	0.01
Erdos	Chemical Shift (PPM)	13.9	21.23/23.28/30.51	37.13/44.37	51.43/58.53	64.99/88.75	165.91/211.57
	Relative area	0.040	0.22	0.12	0.015	0.028	0.018

Kerogen sample		Aromaticity				
		Ortho‐Oxyaromatic protonated $\left[f_{\mathrm{ar}}^{1}\right]$	Ortho‐Oxyaromatic branched $\left[f_{\mathrm{ar}}^{2}\right]$	Aromatic protonated$\left[f_{\mathrm{ar}}^{3}\right]$	Bridging ring junction $\left[f_{\mathrm{ar}}^{4}\right]$/Aromatic Branched $\left[f_{\mathrm{ar}}^{5}\right]$	Oxy‐aromatic $\left[f_{\mathrm{ar}}^{O}\right]$
Songliao	Chemical Shift (PPM)	109.29	119.33	113.99/124.46/128.16	132.74/139.98/147.89/153.83	160.72
	Relative area	0.015	0.039	0.08	0.155	0.008
Erdos	Chemical Shift (PPM)	107.71	121.32	114.96/127.66	133.86/139.32/143.85/149.60	156.23
	Relative area	0.025	0.11	0.20	0.219	0.005

Aromaticity *f*_ar_ reflects the structure of organic matter quantificationally, which is usually directly related with the degree of evolution. Aromaticity can be obtained by the high resolution ^13^C NMR spectra, whose value is the ratio of absorption peak area of the 100–165 ppm segment to the area of 0–220 ppm segment.^39^ The aromaticity degrees ($f_{\mathrm{ar}}^{I}$, $f_{\mathrm{ar}}^{\mathrm{III}}$) of Songliao and Erdos kerogen can be calculated as

(1.a) $$f_{\mathrm{ar}}^{I}=f_{\mathrm{ar}}^{1I}\;+f_{\mathrm{ar}}^{2I}+f_{\mathrm{ar}}^{3I}+f_{\mathrm{ar}}^{4I}+f_{\mathrm{ar}}^{5I}+\;f_{\mathrm{ar}}^{\mathrm{OI}}=\;29.7\%$$

(1.b) $$f_{\mathrm{ar}}^{\mathrm{III}}=f_{\mathrm{ar}}^{1\mathrm{III}}\;+f_{\mathrm{ar}}^{2\mathrm{III}}+f_{\mathrm{ar}}^{3\mathrm{III}}+f_{\mathrm{ar}}^{4\mathrm{III}}+f_{\mathrm{ar}}^{5\mathrm{III}}+\;f_{\mathrm{ar}}^{\mathrm{OIII}}=\;55.9\%$$

Another important parameter of kerogen structure is aliphaticity *f*_al_, which is ratio of area of 0–90 ppm segment (representing the aliphatic carbon) to the area of 0–220 ppm segment. And the aliphaticity degrees are

(2.a) $$f_{\mathrm{al}}^{I}=f_{\mathrm{al}}^{1I}\;+f_{\mathrm{al}}^{2I}+f_{\mathrm{al}}^{3I}+f_{\mathrm{al}}^{O_{1}I}+\;f_{\mathrm{al}}^{O_{2}I}=\;69.3\%$$

(2.b) $$f_{\mathrm{al}}^{\mathrm{III}}=f_{\mathrm{al}}^{1\mathrm{III}}\;+f_{\mathrm{al}}^{2\mathrm{III}}+f_{\mathrm{al}}^{3\mathrm{III}}+f_{\mathrm{al}}^{O_{1}\mathrm{III}}+\;f_{\mathrm{al}}^{O_{2}\mathrm{III}}=\;42.3\%$$

The branching degrees of alkyl chain can be obtained by the ratios of methine and quaternary carbon to aliphatic carbon, which are

(3.a) $${\mathrm{BI}}_{I}=f_{\mathrm{al}}^{3I}/\;f_{\mathrm{al}}^{I}=15.87\%$$

(3.b) $${\mathrm{BI}}_{I}=f_{\mathrm{al}}^{3\mathrm{III}}/\;f_{\mathrm{al}}^{\mathrm{III}}=28.37\%$$

The branching degree of alkyl chain of Erdos kerogen is higher than that of Songliao kerogen, which means the molecule of Erdos kerogen is more compact.

The shift of bridge aromatic carbon is near 131 ppm, and the 130–140 ppm segment represents chemical shift of alkyl substituted aromatic carbon. The contribution of oxygen to the substituted aromatic carbon is in the 155–165 ppm range. Nonprotonated aromatic carbon content is the sum of these three parts. The degrees of aromaticity substitution, which are the ratios of nonprotonated aromatic carbon to total aromatic carbon, can be obtained by the following equations

(4.a) $$\delta_{I}=\left(f_{\mathrm{ar}}^{2I}+f_{\mathrm{ar}}^{4I}+f_{\mathrm{ar}}^{5I}+f_{\mathrm{ar}}^{\mathrm{OI}}\right)/\;f_{\mathrm{ar}}^{I}=68.01\%$$

(4.b) $$\delta_{I}=\left(f_{\mathrm{ar}}^{2\mathrm{III}}+f_{\mathrm{ar}}^{4\mathrm{III}}+f_{\mathrm{ar}}^{5\mathrm{III}}+f_{\mathrm{ar}}^{\mathrm{OIII}}\right)/\;f_{\mathrm{ar}}^{\mathrm{III}}=59.75\%$$

By the degrees, there are 3 to 5 aromatic hydrogen atoms are replaced by substituent groups.

Average aliphatic chain lengths are about

(5.a) $$\lambda_{I}=f_{\mathrm{al}}^{I}/\;f_{\mathrm{ar}}^{5I}\;=\;8$$

(5.b) $$\lambda_{\mathrm{III}}=f_{\mathrm{al}}^{\mathrm{III}}/\;f_{\mathrm{ar}}^{5\mathrm{III}}\;=\;3$$

which indicates the aliphatic chain length of Songliao kerogen is longer than that of Erdos. The carbon skeletons of kerogen molecules are obtained by the analyzing of ^13^C NMR spectra.

#### 2.1.5 Kerogen Macromolecular Models

**Figure 5:**
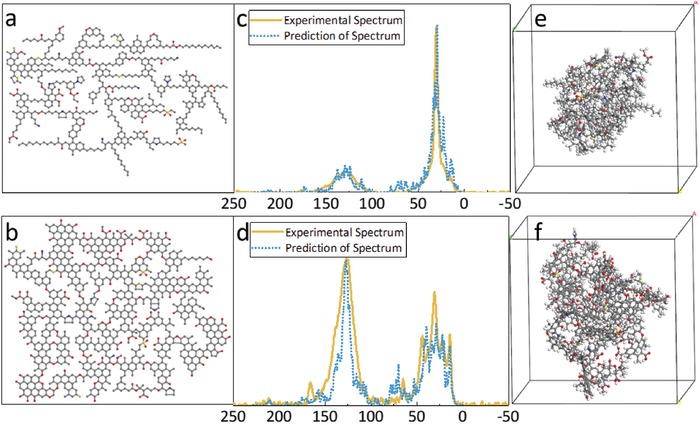
The macromolecular models of kerogen. The 2D kerogen macromolecular models of a) Songliao kerogen and b) Erdos kerogen. Comparison of the experimental ^13^C NMR spectra (yellow lines) and prediction of the ^13^C NMR spectra of the 2D kerogen molecular models (the blue dotted line) of c) Songliao kerogen and d) Erdos kerogen. The 3D kerogen macromolecular models of e) Songliao kerogen and f) Erdos kerogen corresponding to the 2D models. The molecular formulas and molecular weights of Songliao and Erdos are C_612_H_846_N_10_O_40_S_7_ and 9208.06 g mol^−1^, C_853_H_756_N_8_O_115_S_6_ and 13 151.90 g mol^−1^, respectively.

The elemental ratios, the relative contents of the functional groups, and carbon skeletons are obtained by EA, XPS, FT‐IR, and ^13^C NMR. Kerogen is with amorphous structure whose composition and structure are complex. The 2D macromolecular structures (statistical average) are established by setting the aromatic functional groups as the basic elements, distributing heteroatomic functional groups into the basic elements and bounding those groups by aliphatic side chains. The “average molecular structure” referring to a cluster cannot be regarded as a basic unit of any specific molecule in the whole body.^18^ Instead, it is regarded as a mathematical model containing various functional groups and could be used to reflect the typical chemical and physical properties of mixture molecular groups.^19^ As shown in **Figure** **5**a,b, the 2D models of Songliao and Erdos kerogen samples have 29 and 114 aromatics, and the aromatic carbon numbers are 167 and 419, respectively. The ratios of aromatic carbon to aliphatic carbon of both samples are similar to the results of XPS and ^13^C NMR, and that means the elemental compositions and the aromatic degrees of kerogen models are in good agreement with the experimental results.

In order to verify the rationality of kerogen 2D molecular models, MestReNova software suite was used to calculate the ^13^C NMR spectra of the molecular models and compare them with the experimental spectra. As shown in Figure 5c,d, the fitting peaks of models were obtained by giving a linewidth to each chemical shift of the carbon atom, and assuming each peak of the ^13^C NMR simulation spectra is Gauss peak model. The superposition of the fitting peaks of the model is the simulated peak. And the simulated NMR spectra fairly correspond with the experimental spectra, by which the rationality of the kerogen molecular models used for the following studies is further verified.

### 2.2 3D Molecular Constructions by MD Simulations

Based on the 2D kerogen models, the 3D macromolecular models of kerogen with abundant and comprehensive functional groups were established. First, the geometric structures were modified to eliminate the irrational structure, so that the bonds, the bond angles, and the angles of torque are with chemical rationality. Then preliminary optimizations of the structures were by geometry optimization of MM to obtain the optimal geometric configuration. The smart optimization was chosen in preliminary optimization, which conjugates the steepest descent method, the gradient method, and the Newton method. While the macromolecules optimized by MM always are with local minimum energy, rather than the global minimum of the potential energy surfaces. The annealing dynamics simulations were used to optimize the model structures, to overcome the shortcoming and find out the global energy minimum.^20^ Annealing in the taskbar of the Forcite module was chosen for the annealing dynamics. The initial temperature and the mid‐cycle temperature of annealing kinetics simulations were set as 300 K and 600 K, respectively. The canonical (NVT, constant number of atoms, volume, and temperature) ensemble was chosen in MD simulations. The simulation times are 300 ps. The Nose–Hoover method of temperature control program was selected, as this method can couple any number of atoms with a hot bath together and eliminate local associated motion to simulate the temperature fluctuation of macro system. At last, the output structures of each annealing dynamics cycle were performed MM calculation to ensure they are in low energy state. The final outputs of the least energy configuration are selected as the optimal geometric configurations (Figure 5e,f).

### 2.3 Analysis of Chemical Kinetics of Kerogen Pyrolysis by ReaxFF Simulations

The NVT ensemble was selected to calculate the reactions of hydrocarbon generation from kerogen. The ReaxFF module of Amsterdam Modeling Suite software was used for the simulations, to study the effect of temperature on the pyrolytic products. HCONSB.ff was chosen as the force field. The Songliao and Erdos kerogen molecules were restricted in the 60 × 60 × 60 lattices and the simulations were calculated with periodic boundary conditions (PBC) using 0.25 fs as the time step.

**Figure 6:**
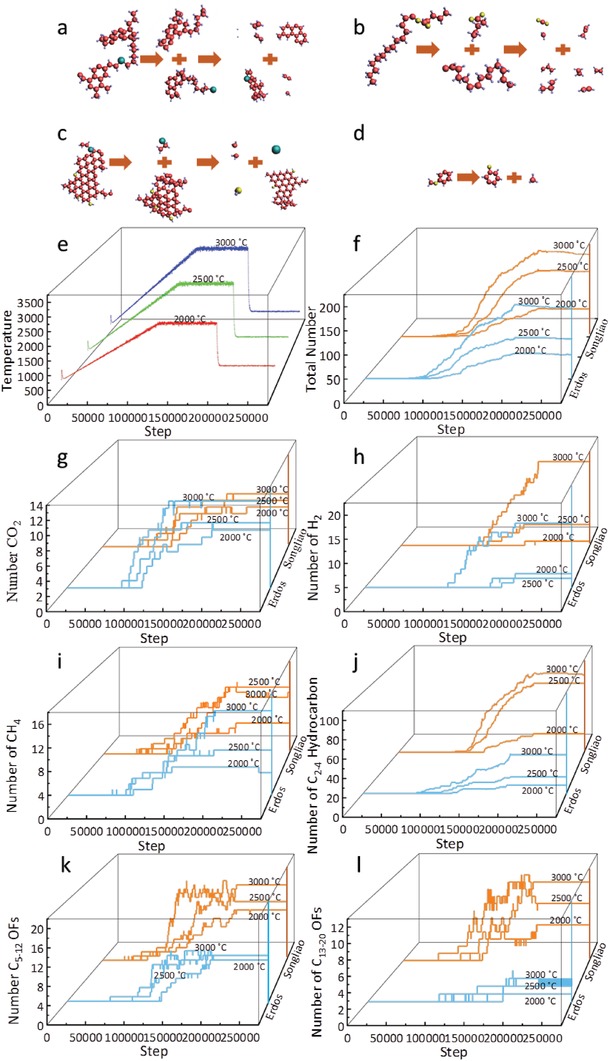
The pyrolysis process under different temperature control. a–d) The typical pyrolysis reactions of kerogen structures. e) The temperatures of simulations. f–l) Dynamic changes of the number of kerogen pyrolytic products accompanied by pyrolysis of Songliao and Erdos kerogens. The OFs is the abbreviation of organic fragments.

The compositions of pyrolytic products of 3D model simulations were analyzed under different heating conditions as shown in **Figure** **6**c. Kerogen can be resolved to light hydrocarbons (CH_4_, C_2_H_4_, etc.), heavy hydrocarbons, molecules containing heteroatoms, and inorganic small molecules (CO_2_, H_2_, etc.). To facilitate analysis, the pyrolytic products are divided into CO_2_, C_1_ hydrocarbon fragments, C_2_–C_4_ hydrocarbon fragments, C_5_–C_12_ molecular fragments, and C_13_–C_20_ molecular fragments. The gaseous hydrocarbons (C_1_–C_4_) are the main components of shale gas in shale reservoirs in which hydrocarbons of C_2_–C_4_ molecules are named as heavy hydrocarbon gas. The organic molecules of C_5_–C_12_, which boil between 30 °C and 200 °C, belong to naphtha.^40^, ^41^ And the diesel‐fuel is from C_13_ to C_20_.

The results of simulations are shown in Figure S1 in the Supporting Information, which show the pyrolysis generates more pyrolytic products and is more complete at higher temperature, and Songliao kerogen is more easily pyrolysis into small molecular fragments. The typical pyrolytic process, pyrolytic temperatures, total fragments number ,and pyrolytic products numerical evolutions are shown in Figure 6. Kerogen pyrolysis products are related to the bond energy of kerogen molecules. The mechanism of typical reaction of kerogen pyrolysis was shown in Figure 6a–d. The fused aromatic rings do not undergo cracking, while dehydrogenation occurr in the six‐membered rings. The long chain hydrocarbons will be cracked into small hydrocarbon fragments. As shown in Figure 6b, the C–C(O) band energy is higher than the C(O)–O band energy, and the reaction eventually has produced CO_2_. The benzene–oxygen bond is harder to be broken than methyl–oxygen bond as shown in Figure 6d, because the dissociation energy of benzene–oxygen bond (356 kJ mol^−1^) is higher than that of methyl–oxygen bond (247 kJ mol^−1^), as a result many oxygen atoms in Erdos kerogen are at the end of the fused aromatic ring. The total number of products increases with temperature and the number of pyrolytic products of Songliao kerogen is higher than that of Erdos at the same temperature. That means the rate of pyrolytic reaction is higher at high temperature.

The numbers of pyrolytic products obtained by simulations are listed in **Table** **4**. At 2500 and 3000 °C, CO_2_ numbers of Erdos kerogen are more than numbers of Songliao. The methane (CH_4_) number of Erdos kerogen increases rapidly from 2500 to 3000 °C. And it is more than that of Songliao at 3000 °C. That means Erdos kerogen generates more CH_4_ when the pyrolysis is more complete. The naphtha and diesel‐fuel numbers of Erdos are less, which illustrates that Songliao kerogen is with better oil potential and proves the rationality of the kerogen molecular models from another side. The increased number of pyrolytic of Songliao kerogen simulations from 2000 to 2500 °C is more than that of simulations from 2500 to 3000 °C, while number of Erdos kerogen rises more significantly in the simulations from 2500 to 3000 °C. The results of the simulations show that the number of small molecules produced by pyrolysis does not increase linearly with the increase of temperature.

**Table 4:** The quantitative contents of pyrolytic products

Simulation		CO_2_	C_1_	C_2_–C_4_	C_5_–C_12_	C_13_–C_20_	Total number
Songliao	2000 °C	8	6	22	12	5	67
	2500 °C	6	13	82	14	11	156
	3000 °C	9	12	92	18	8	198
	Ratio	1:0.75:1.125	1:2.17:2	1:3.73:4.18	1:1.17:1.5	1:2.2:1.6	1:2.33:2.96
Erdos	2000 °C	7	4	9	9	1	53
	2500 °C	8	8	18	10	2	87
	3000 °C	12	15	42	10	2	154
	Ratio	1:1.14:1.71	1:2:3.75	1:2:4.67	1:1.11:1.11	1:2:2	1:1.64:2.91

**Figure 7:**
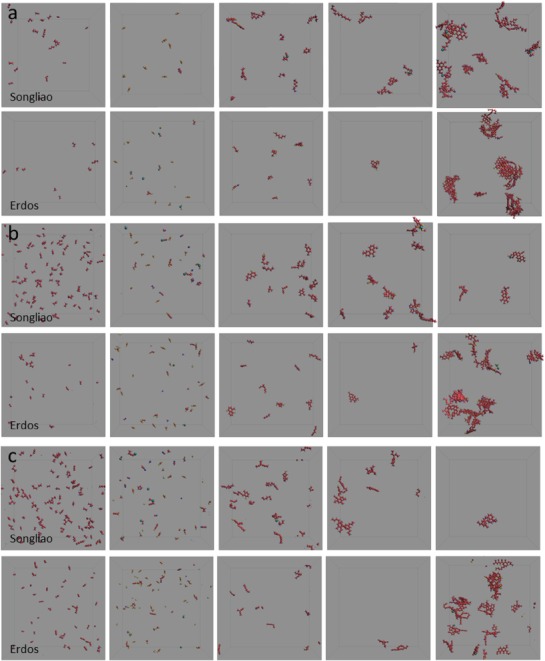
The pyrolytic products of kerogen. Column 1 to 6 represent C_2_–C_4_ hydrocarbon fragments, C_1_–C_4_ organic heteroatomic fragments and inorganic molecules, C_5_–C_12_ organic fragments, C_13_–C_20_ organic fragments and C_20+_ organic fragments in the pyrolytic products at a) 2000 °C, b) 2500 °C, and c) 3000 °C, respectively.

The structures of pyrolytic fragments (C_2_–C_4_ hydrocarbon fragments, inorganic molecules and C_1_–C_4_ organic heteroatomic fragments, C_5_–C_12_ organic fragments, C_13_–C_20_ organic fragments, and C_20+_ organic fragments) are shown in **Figure** **7**. At 3000 °C, none of O, S, and N atoms of Songliao kerogen is distributed in C_20+_ organic molecule, and about 94% O, 86% S, and all of N is in small organic (C_2_–C_4_) and inorganic molecules, while Erdos kerogen is with about 34% O, 50% S, and 63% N in C_20+_ organic molecule. Heteroatoms containing in or bonding to fused aromatic rings may contribute to this phenomenon. Erdos kerogen is with more large molecular fragments remaining, as Erdos kerogen is rich in fused aromatic rings whose C–C bonds are with high energy.

**Figure 8:**
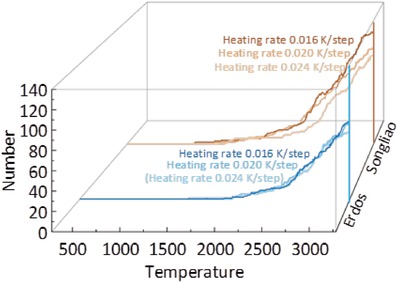
The total numbers of pyrolytic products are obtained by the simulations with 0.016 K, 0.020, and 0.024 K per step. The yellow lines represent the numbers of Songliao kerogen and the color darkens gradually as the heating rate decreases.

In order to reveal the influence of the heating rates, the simulations with the same final temperature of different rates were performed. The simulations with 0.016, 0.02, and 0.024 K per step rates were set as the same final temperature 3000 °C. The total number of pyrolytic products of Songliao and Erdos kerogen molecules are shown in **Figure** **8**. The slower the heating rate is, the higher the efficiency of pyrolysis becomes. The temperature of Songliao kerogen at which the pyrolytic reaction occurs is lower than that of Erdos kerogen. The change of heating rates has less effect on the pyrolytic reaction rate of the Erdos kerogen than that of Songliao by Figure 8. And this phenomenon offers yet another confirmation that the Erdos kerogen model is more stable than Songliao kerogen in the pyrolysis process.

### 2.4 Comparison of the Pyrolysis between the Hybrid MD/fbMC Approach and Experimental Results

**Figure 9:**
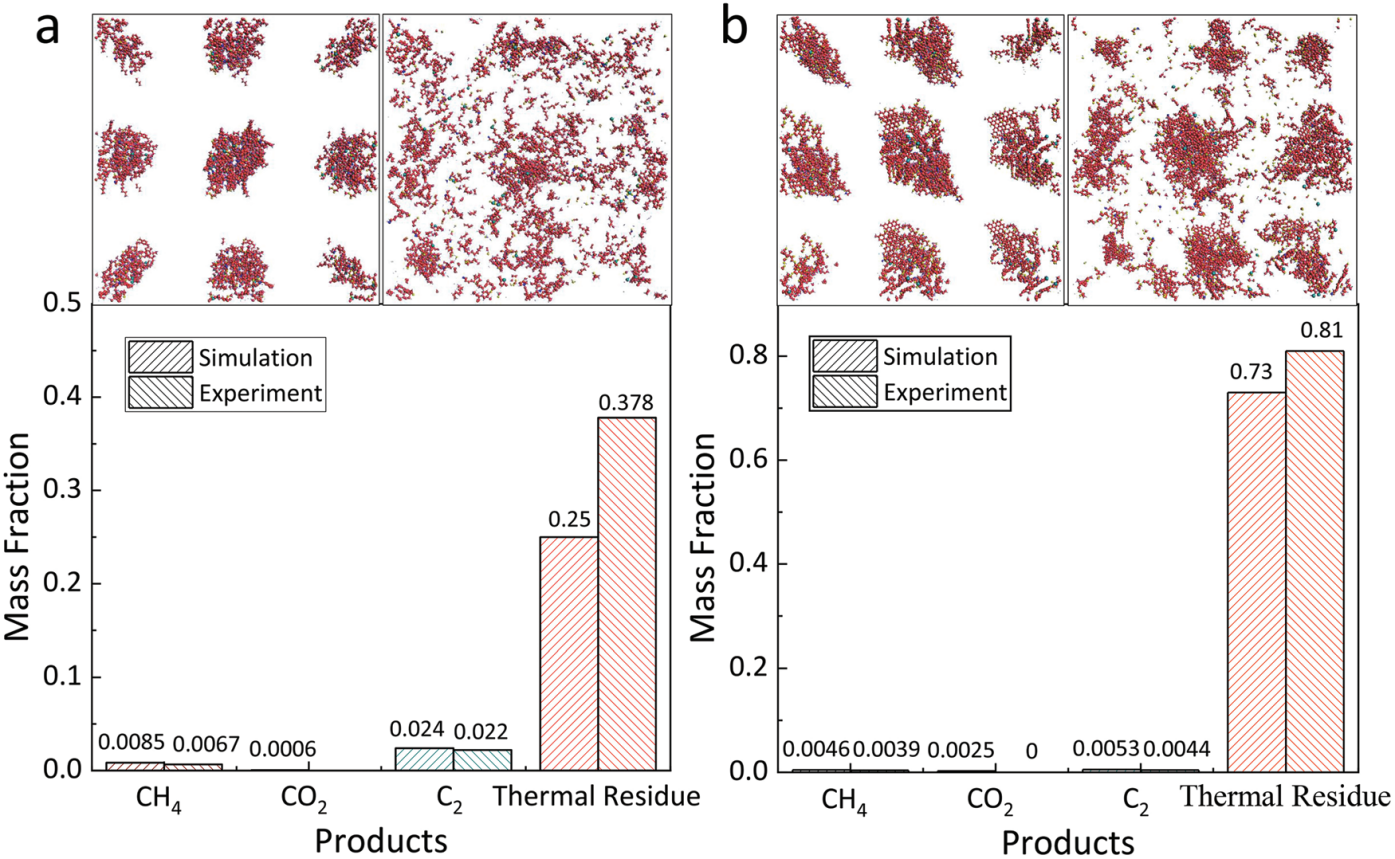
The supercell of kerogen model, pyrolytic products of kerogen and the mass fractions of gases and thermal residue of a) Songliao kerogen and b) Erdos kerogen using hybrid MD/fbMC approach, respectively. The columns filled with bivariate diagonal represent the mass fractions obtained by the experiments and the columns with leading diagonal are mass fractions of simulations.

While the temperature of ReaxFF simulation is higher than the experimental temperature of pyrolysis in order to start reacting in computational time, by means of hybrid simulations coupled MD to fbMC, the slow chemical reaction in macro timescale is obtained in micro timescale.^42^ Similarly, the kerogen molecules were restricted in the 60 × 60 × 60 lattices by which 2 × 2 × 2 supercells are generated to get the statistical averages of pyrolytic products. The frequency of fbMC steps and number of fbMC steps were set as 10 000 steps and the heating rate was set as 0.65 K per step, using 0.25 fs as the time step. The force field of ReaxFF was consistent with the previous simulations. According to the preliminary experiments of PY‐GC/MS, Songliao kerogen is pyrolyzed at 800 °C and pyrolytic gases can be detected by the gas detector, while pyrolytic gases of Erdos kerogen are detected at 1100 °C. Therefore, the simulation temperatures of Songliao and Erdos kerogen were set as 800 and 1100 °C, respectively. The calculations were with 400 000 time steps (0.1 ns) and the pyrolysis results have been shown in **Figure** **9**.

The simulations of kerogen pyrolysis by hybrid MD/fbMC approach are carried out at the experimental temperatures. According to the experiments, the amounts of substance of CH_4_, CO_2_, C_2_H_4_, and C_2_H_6_ of pyrolytic generation are obtained. The results of experiments and simulations of hybrid MD/fbMC are shown in Figure 9. The CH_4_, C_2_H_4_, and C_2_H_6_ of Songliao kerogen by pyrolysis are more than those of Erdos by experiments and simulations, which is consistent with the simulations before. The numbers of pyrolysis gases (CO_2_, CH_4_, C_2_H_4_, and C_2_H_6_) are obtained by analyzing the simulation results. The masses of C_23+_ of simulations are calculated to compare with the residual masses of experiments. The residual mass of Erdos kerogen is much higher than that of Songliao kerogen, which is consistent with the previous result that Erdos kerogen is more difficult to be pyrolyzed into small molecules. According to the results of simulations and experiments, mass fractions of gases and residuals can be calculated. The mass fractions of gases by simulations are slightly higher than the experimental results, while that of thermal residue lower than the experimental results. Some mineral impurities of the kerogen samples may contribute to this phenomenon. The mass fraction of thermal residue is chosen as the standard to calculate the impurities mass by (*m*_macro_ + *m*_min_)/*m*_sample_ = ω_res‐expri_  and *m*_macro_/(*m*_sample_ − *m*_min_) = ω_res‐simu_, where *m*_macro_, *m*_min_, *m*_sample_, ω_res‐expri_ and ω_res‐simu_ are the mass of pyrolytic products of residue, mass of mineral impurities, mass of sample, mass fraction of thermal residue by experiments, and mass fraction of thermal residue to compare with simulations. The mass of mineral impurities of Songliao and Erdos kerogens are 0.085 and 0.14 mg. Then the mass fractions of CH_4_, C_2_ hydrocarbon of Songliao kerogen are about 0.081 and 0.026, those of Erdos are 0.0054 and 0.0061. The simulation results are very similar to the experimental results.

## 3 Conclusions

We establish the kerogen macromolecules with abundant chemical information that are large enough to contain the functional groups presenting at low occupancy. The reliability of the molecules is verified, and the 3D molecular structures with global energy minimization are established.

By analyzing the pyrolytic process of the 3D kerogen models and the effects of temperature and heating rate on the pyrolysis of kerogen, the chemical kinetics of kerogen pyrolysis is clarified. Bond energy is the main reason that affects pyrolysis components and processes. The typical reaction mechanism of kerogen pyrolysis is found out, containing long chain hydrocarbons cracking (reaction of shale gas generation), dehydrogenation of six‐membered rings and –C(O)–C to be CO_2_. The pyrolysis of Erdos kerogen is less affected by the rate of heating and produces fewer small molecules. Therefore, the kerogen molecule rich in fused aromatic rings is more stable, and the heteroatoms in or bonding to fused aromatic rings are distributed in large molecular structures. Erdos kerogen (rich in fused aromatic rings) generates less shale oil than Songliao kerogen, while it can produce more CH_4_ at high temperature. And the quantitative relationships of CH_4_, heavy hydrocarbon gases, naphtha and the diesel‐fuel obtained by kerogen pyrolysis of different mining areas are established at different temperature control conditions.

To carry out the simulations at experimental temperature in micro time scale, the hybrid MD/fbMC approach was used to simulate the pyrolysis of kerogen. The mass fractions of CH_4_, other pyrolytic gases and residues of simulations are consistent with those of PY‐GC/MS. The effectiveness and superiority of the simulation method are verified. Our findings may help to understand the oil/gas generation from the atomic level and assist in the future studies of kerogen mechanical property and oil/gas transport in micro‐nanopores.

## 4 Experimental Section

*Kerogen Extraction*: Kerogen samples were extracted from shale to study structure and properties of kerogen. Shales were crushed and fully expanded, which lie about 3000 m underground in Erdos and 400 m underground in Songliao basin. Using GBT 19144‐2010 method (Isolation Method for Kerogen from Sedimentary Rock), Kerogen samples were extracted by removing inorganic minerals through a series of chemical and physical methods. Hydrochloric acid solution (HCl) was used repeatedly for the carbonate removal, then HCl and hydrofluoric acid (HF) mixed solution was employed to remove silicate repeatedly. Sodium hydroxide (NaOH) was selected to remove silicon dioxide (SiO_2_) further after taking out the centrifugal supernatant. HCl and zinc powder were added into samples repeatedly to remove pyrite. Then the halide ions were removed by eluting the remnants with water. After centrifugation, the kerogen samples were frozen and dried, the soluble organic matter was cleaned by chloroform, and the loss on ignition was measured. And pyrite treatment was carried out repeatedly to obtain relatively pure kerogen samples, until the loss of the extract was more than 75%.

*Experiments of Molecular Construction*: First, XPS (ESCALab220i‐XL) was performed to analyze chemical states of elements of samples. A computation multipeak resolution method (using XPSPEAK41) was applied for the XPS intensity curves of regional XPS scans of C 1s, O 1s, N 1s, S 2p of kerogen. C, O, and N (1s) do not appear the energy level splitting, so a chemical state is with the single peak in the XPS spectra. The chemical states of S appear in the form of doublet of 2p_1/2_ and 2p_3/2_ (Area_2p1/2_:Area_2p3/2_ = 1:2), since the 2p layer electrons of S generate energy‐level splitting due to the spin‐orbit coupling under the excitation of X‐rays.^43^ However, XPS cannot obtain the H elemental relative content as H is with just one electronic shell, so EA was performed to determine the atomic ratio of H. The results of XPS and EA were applied to establish the molecular formulas. Then, the FT‐IR spectroscopy measurement was used to study the compositions and contents of kerogen. Kerogen samples were mixed 1:100 with the potassium bromide (KBr) to obtain the FT‐IR transmittance spectra using pure KBr as background. The functional groups of kerogen molecules and relative abundances can be representation by FT‐IR spectrum. The main absorption frequency of each group and the vibration characteristics are shown in Table S1 in the Supporting Information.^38^ At last, cross‐polarization magic angle spinning carbon‐13 nuclear magnetic resonance spectrum technology was performed to obtain the relative contents between aromatic, aliphatic, and carbonyl carbon, in order to study on the kerogen carbon skeleton. The experiments on samples were completed on BRUKER AVANCE III 400 M using 8 KHz rotation speed and adamantane (ada) as standard substance.

*Pyrolytic Experiments*: The heating rate of experiments being performed by PY‐GC/MS to trap gases of H_2_, CO_2_, CH_4_, C_2_H_6_, and C_2_H_4_ was set as 20 000 K s^−1^ to effectively avoid secondary cracking. The 0.5 mg samples of Songliao and Erdos kerogens were maintained at 800 °C and 1100 °C for 20 s, respectively.

## Conflict of Interest

The authors declare no conflict of interest.

## Supporting information

Supplementary
