# Supplementary material for: The Constructions and Pyrolysis of 3D Kerogen Macromolecular Models: Experiments and Simulations
Source: Glob Chall. 2019 Apr 7;3(5):1900006. doi: 10.1002/gch2.201900006 (PMC6498132; doi:10.1002/gch2.201900006)
Supplement: Supplementary file 1 — Supplementary [file GCH2-3-1900006-s001.pdf]

Copyright WILEY-VCH Verlag GmbH & Co. KGaA, 69469 Weinheim, Germany, 2019.

Global Challenges
Open Access

Supporting Information

for *Global Challenges,* DOI: 10.1002/gch2.201900006

The Constructions and Pyrolysis of 3D Kerogen Macromolecular Models: Experiments and Simulations

*XiaoHe Wang, XianFu Huang, Kui Lin, and Ya-Pu Zhao**

Copyright WILEY-VCH Verlag GmbH & Co. KGaA, 69469 Weinheim, Germany, 2018.

Supporting Information

The Constructions and Pyrolysis of 3D Kerogen Macromolecular Models: Experiments and Simulations

*XiaoHe Wang, XianFu Huang, Kui Lin and Ya-Pu Zhao**

Table S1. The bond position corresponding to the functional group.

Group	Band position (cm^{-1})	Assignment
Hydroxyl	3400-3200	-OH (stretching)
	1300-1000	C-O (stretching)
	769-659	-OH (bending vibration)
Aromatic	3100-3010	Aromatic C-H (stretching)
	1600-1450	Phenyl Ring Substitution Bands (Ar-R)
	880-680	Phenyl Ring Substitution Overtones
Alkanes	3000-2850	Alkanes C-H (stretching)
	1465-1340	Alkanes C-H (scissoring and bending)
Ether	1300-1000	C-O (stretching)
	1150-1060	aliphatic ether (stretching)
	1270-1230	Ar-O (stretching)
	1050-1000	R-O (stretching)
aldehydes/ketones	1750-1700	aldehydes C=O (stretching)
	2820,2720	aldehydes C-H/ketones C=O (stretching)
Carboxylic	3300-2500	-OH(stretching)
	1720-1706	C=O (stretching)
	1320-1210	C-O (stretching)
	920	Ester -OH (bending)
Aldehyde	1750-1735	C=O(stretching)
	1210-1163	C-C(=O)-O (stretching)
Amidogen	3500-3100	N-H (stretching)
	1350-1000	C-N (stretching)
	1640-1560	N-H (bending)
	900-650	N-H (bending)

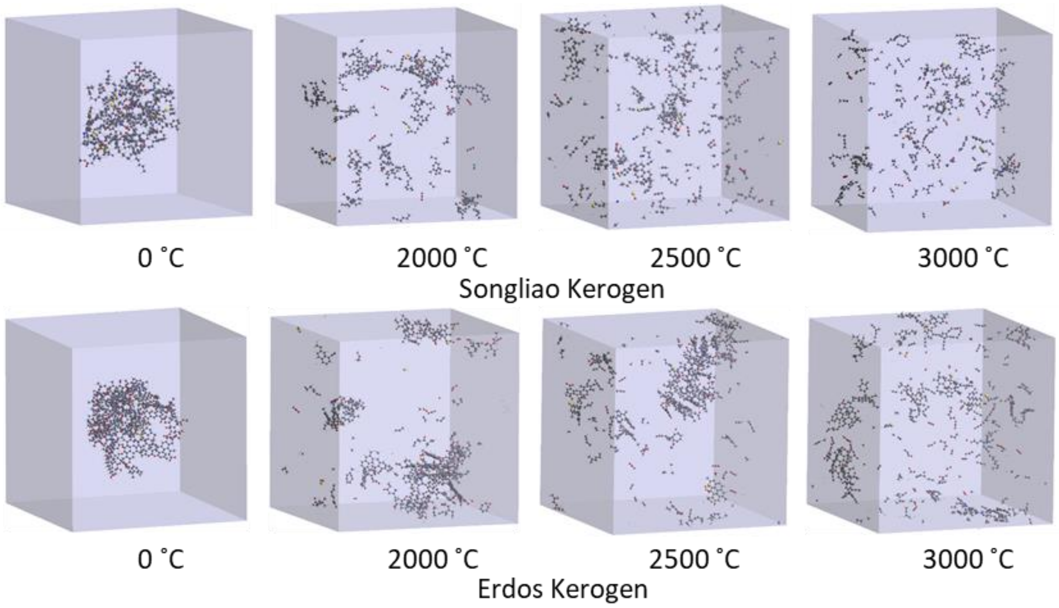

Figure S1. Pyrolytic products of kerogen obtained under different temperature control conditions.

Supplementary Note 1

The Functional Group Assignment of FT-IR. The functional group region of 4000-1300 cm^{-1} is used in the identification of functional groups, which is stretching vibration absorption band (sparser and easy to identify). There are deformation bands due to bending vibration in 1300-4000 cm^{-1}, in addition to the stretching vibration of a single band. This vibration is related to the structure of the whole molecule. When the molecular structure is slightly changed, there are subtle differences in the absorption of the region and show the molecular characteristics. This situation is like human fingerprints, so called fingerprint zone. The fingerprinting region is useful for identifying compounds of similar structure and can serve as a by-product of the presence of a radical group of compounds.

Supplementary Note 2

The Method of 3D Molecular Models Construction. The 3D molecular models were constructed using molecular mechanics (MM), to further study the kerogen pyrolysis. As Forcite module of material studio can be used for reliable geometric optimization for molecular or periodic systems, the 3D structures optimization was completed by MS.Forcite. The Dreiding force field was used in the future simulation process. The Dreiding force field has been selected because it can be applied to the studies of small organic molecules, macromolecules, and structure of main group elements. The Dreiding force field can be expressed as:

$$
\begin{aligned}
E &= \underbrace{E_{\text{bond}} + E_{\text{angle}} + E_{\text{tor}} + E_{\text{inver}}}_{\text{valence energy}} + \underbrace{E_{\text{van}} + E_{\text{elec}} + E_{\text{hydr}}}_{\text{non-bond energy}} \\
&= \sum_{\text{bond}} \frac{K_b}{2}\left(b-b_0\right)^2 + \sum_{\text{angle}} \frac{K_\theta}{2}\left(\cos\theta-\cos\theta_0\right)^2 + \sum_{\text{dihedral}} \frac{K_\phi}{2}\left[1-\cos n\left(\phi-\phi_0\right)\right]^2 , \\
&\quad + \sum_{\text{inversion}} E_\chi\left(\cos\chi\right) + \sum_{\text{nonbond}} \left\{ \varepsilon_{ij}\left[\left(\frac{\sigma_{ij}}{r}\right)^{12} - 2\left(\frac{\sigma_{ij}}{r}\right)^{6}\right] + \frac{q_i q_j}{r} \right\}
\end{aligned}
\tag{S.1}
$$

where $E_\chi\left(\cos\chi\right) = K_\chi\left(\cos\chi - \cos\chi_0\right)^2$, the valence energy contains bond energy E_{bond}, angle energy E_{angle}, torsion energy E_{tor} and inversion energy E_{inver}, while non-bond energy is constituted by van der Waals energy E_{van}, electrostatic energy E_{elec} and hydrogen bond energy E_{hydr}.[S1]

Supplementary Note 3

The Method of Pyrolytic Simulations. The reaction field method has been successfully applied to research of organic macromolecule system. In this study, ReaxFF was used to explore pyrolytic process of kerogen at the high temperature and analyze the main products of kerogen pyrolysis from the atomic level, which has applied to simulate the pyrolysis of dimethyl siloxane polymer successfully.[S2] Core of ReaxFF is using BO_{ij} which is defined as a function of bond order for expressing the energy terms and interaction between atoms, through the function calculation is divided into bond, angle, and dihedral angle, Coulomb, van der Waals and conjugate adjustment terms, etc.[S3] Each part of the intramolecular energy is expressed through the bond level, except the non-bond interactions. The general form of the force field is

$$
\begin{aligned}
E &= E_{\text{bond}} + E_{\text{lp}} + \underbrace{E_{\text{over}} + E_{\text{under}}}_{\text{atom over-/under-coordination}} + \underbrace{E_{\text{val}} + E_{\text{pen}} + E_{\text{coa}}}_{\text{valence angle terms}} \\
&\quad + E_{\text{tors}} + E_{\text{conj}} + E_{\text{Hbond}} + \underbrace{E_{\text{vdW}} + E_{\text{Coulomb}}}_{\text{non-bonded energy}}
\end{aligned}
\tag{S.2}
$$

Terms of the Equation are bond energy E_{bond}, lone-pairs bond energy E_{lp}, penalty energy of atom over-/under-coordination $E_{\mathrm{over}}/E_{\mathrm{under}}$, valence angle energy E_{val}, energy penalty for handling atoms with two double bonds E_{pen}, coalition (three-body conjugation) energy E_{coa}, torsion angle energy E_{tors}, conjugation bonds energy E_{conj}, hydrogen bond energy E_{Hbond}, nonbonded van der Waals interactions energy E_{vdW} and Coulomb interaction energy E_{Coulomb}, respectively.[S1]

References

S1. Y. P. Zhao, *Physical mechanics of surfaces and interfaces*, Science Press, Beijing, China **2012**.

S2. C. Zhan, C. Lian, Y. Zhang, M. W. Thompson, Y. Xie, J. Wu, P. R. Kent, P. T. Cummings, D. e. Jiang, D. J. Wesolowski, *Adv. Sci.* **2017**, *4*, 1700059.

S3. K. Chenoweth, A. C. van Duin, S. Dasgupta, W. A. Goddard, *J. Phys. Chem. A* **2009**, *113*, 1740.
